# NLRP3: A Novel Mediator in Cardiovascular Disease

**DOI:** 10.1155/2018/5702103

**Published:** 2018-04-08

**Authors:** Wenyi Zhou, Chunyuan Chen, Zhiheng Chen, Lin Liu, Jie Jiang, Zhixiang Wu, Mingyi Zhao, Yanfang Chen

**Affiliations:** ^1^Cardiovascular Department, The Second Affiliated Hospital of Guangzhou Medical University, Guangzhou Institute of Cardiovascular Disease, Guangzhou 510000, China; ^2^Guangdong Cardiovascular Institute, Guangdong General Hospital, Guangdong Academy of Medical Sciences, Guangzhou 510100, China; ^3^Department of Pediatrics, The Third Xiangya Hospital, Central South University, Changsha 410013, China; ^4^Department of Pharmacology and Toxicology, Boonshoft School of Medicine, Wright State University, Fairborn, OH 45435, USA

## Abstract

Cardiovascular disease is a major cause of death worldwide. Inflammasome infiltration has been identified to play a central role in the pathological progression of certain cardiovascular diseases, such as vascular damage spanning atherosclerosis, aneurysm, or arteritis; ischemic heart disease; and other nonischemic heart diseases including diabetic cardiomyopathy, chronic heart failure, and hypertension- or virus-induced cardiac dysfunction. The NLRP3 inflammasome, a key participant in the innate immune response, requires both priming and activation signals for the initiation of inflammation. Piling evidence has revealed that the NLRP3 inflammasome could exert an inflammatory effect by inducing the secretion of proinflammatory cytokines (i.e., IL-1*β*, IL-18) or could cause pyroptosis, a novel programmed cell death process, in a caspase-1-dependent manner. The importance of the NLRP3 inflammasome in cardiac disease has been broadly investigated. In this review, we present the current knowledge regarding the function of NLRP in vascular disease, ischemic heart disease, and nonischemic heart disease and discuss the potential therapeutic options targeting the NLRP3 inflammasome.

## 1. Introduction

Cardiovascular disease remains prevalent in both developing and developed countries, presenting high morbidity and mortality rates. Vascular damage such as atherosclerosis, aneurysm, and following ischemic heart diseases accounts for the majority of cardiovascular diseases. Atherosclerosis is a chronically progressive disease characterized by abnormal lipid deposition in large arteries and obstructed blood flow, followed by possible plaque rupture, which could cause embolism in vital organs such as the brain, heart, and kidney [1]. In the last three decades, mounting evidence has supported the notion that inflammation contributes to the pathological process of atherosclerosis in pivotal ways [2]. The initial step of atherosclerosis involves the expression of adhesive factors by vascular endothelial cells and the subsequent accumulation of monocytes and lymphocytes. The next step comprises foam cell formation, a critical feature in atherosclerotic plaque. In this process, monocytes differentiate into macrophages, swallow abundant lipoproteins, and finally eat themselves to death. Under the action of proinflammatory cytokines, atherosclerotic plaque is susceptible to rupture and embolization [2]. Taken together, inflammation participates in every step of atherosclerosis. When embolism occurs in the coronary arteries, the patient is likely to have a heart attack owing to a disrupted blood supply. However, the role of inflammation in myocardial ischemia and reperfusion is rather complicated [3]. Neutrophils have been found to be recruited to the ischemic and reperfused myocardium. Neutrophils can exert harmful effects by secreting proteolytic enzymes, whereas macrophages can exhibit beneficial effects such as promoting angiogenesis and cell proliferation [3]. Moreover, cardiac dysfunction often arises at later stages of metabolic disease. For example, diabetic cardiomyopathy (DCM), characterized by loss of cardiomyocytes and dysfunction of the left ventricle, is a major contributor to the fatality of patients with diabetes [4]. Interestingly, Luo et al.'s work could help us better understand DCM from an inflammatory perspective [4].

The innate immune system, the first line of defense in the human body, responds to stimuli such as infection or danger signals released from cells. Pattern recognition receptors (PRRs) expressed by macrophages, neutrophils, and other inflammatory cells of the innate immune system can recognize these danger signals, which can be divided into two categories: pathogen-associated molecular patterns (PAMPs) and damage/danger-associated molecular patterns (DAMPs) [5]. PRRs are classified into five families: toll-like receptors (TLRs), nucleotide-binding and oligomerization domain- (NOD-) like receptors (NLRs), retinoic acid inducible gene-I- (RIG-I-) like receptors (RLRs), C-type lectins (CTLs), and absent-in-melanoma- (AIM-) like receptors (ALRs) [6]. TLR is the first identified PRR and functions as a transmembrane receptor. Although NLRs resemble TLRs, they function as cytoplasmic receptors instead [6]. Proteins from the NLR family primarily consist of three parts: the NOD (NACHT), the C-terminal leucine-rich repeat (LRR) domain, and the N-terminal effector domain [6, 7]. Based on different types of N-terminal domains, a major site responsible for binding to other proteins, the NLR family is further divided into four groups: NLRs containing the acidic transactivation domain (named NLRA), the baculoviral inhibitory repeat-like domain (named NLRB), the caspase activation and recruitment domain (CARD; named NLRC), and the pyrin domain (named NLRP) [6–8]. The biochemical properties of NLRs have been discussed in detail by MacDonald et al. [8]. The NLRP family comprises 14 members, which mainly participate in inflammasome formation [6]. In this review, we present the link between the NLRP subfamily and cardiovascular disease, with a focus on NLRP3, the most widely investigated member.

## 2. NLRP and NLRP Inflammasomes

The NLRP subfamily, characterized by a pyrin-containing domain in the NLRs, is primarily involved in inflammasome formation [6, 7]. According to Amin et al. [7], an inflammasome complex is generally composed of three parts: sensor receptors that could be triggered by PAMPs or DAMPs, adaptors that could facilitate an inflammatory reaction, and effectors that could initiate the inflammatory cascade. Most members of the NLRP family are equipped with a pyrin domain, which serves to interact with another pyrin domain in apoptosis-associated speck-like protein containing a CARD (ASC), a NACHT domain, and an LRR domain, suggested to function as a PAMP-sensing detector [8]. When a PAMP or DAMP is detected by NLRPs, the pyrin domain in the NLRPs binds to the pyrin domain in ASC, which later combines with procaspase-1 via a CARD-CARD interaction. Subsequently, procaspase-1 is converted into caspase-1, and the inflammasome induces either cytokine secretion of IL-1*β* and IL-18 or pyroptosis (a newly defined programmed cell death) [7]. The release of IL-1 requires two steps: a priming step known to modulate the transcription of pro-IL-1*β* and NLRP3 (signal 1) and an activation step known to assemble the NLRP3 inflammasome complex, which help process pro-IL-1*β* into mature IL-1*β* (signal 2) [5]. Moreover, researchers had discovered that caspase-8 and caspase-1 show similar effects in IL-1*β* conversion [7]. However, two members in the NLRP subfamily are structurally different from the others. NLRP10 does not possess an LRR domain and hence probably plays a part in signaling rather than in sensing; NLRP1 possesses an extra CARD, which enables it to directly bind to procaspase-1 without any involvement of ASC [6]. Apart from the well-known inflammasome-dependent proinflammatory function, Willingham et al. proved that NLRP3 facilitated macrophage necrosis and release of high-mobility group box 1 protein (HMGB1), another proinflammatory factor, in response to pulmonary infection [9]. Moreover, attenuated inflammation was observed in NLRP3-deficient mice, along with a declined survival rate, suggesting a protective role of NLRP3 [9].

**Figure 1:**
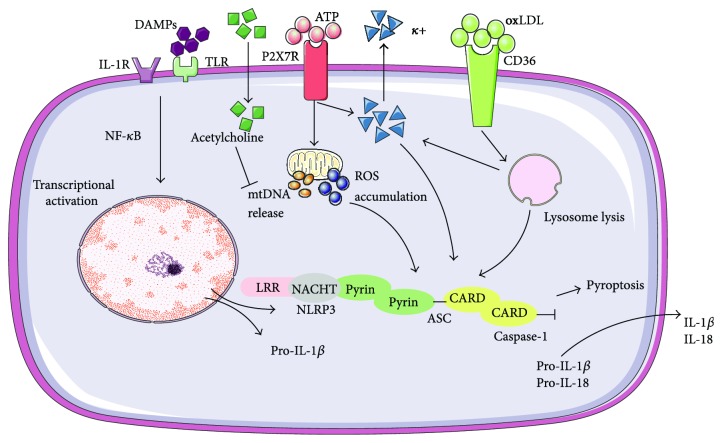
Overall scheme of NLRP3 inflammasome activation.

With regard to the regulation of NLRP3 activation in atherosclerosis, this issue can be discussed in two respects: the priming step and the activation step. Duewell et al. revealed cholesterol crystals to be an activator of the NLRP3 inflammasome in macrophages, probably via induction of lysosomal damage. Oxidized LDL (oxLDL) was also demonstrated to promote atherosclerosis, since it facilitated cholesterol crystallization, induced NLRP3 and pro-IL-1*β* transcription, and thus acted as both signals 1 and 2 [10]. Similar to oxLDL, IL-1*β*, TNF-*α*, and other stimuli recognized by TLRs could serve as priming factors and further induce NLRP3 and pro-IL-1 transcription via the NF-*κ*B pathway [1]. Except for cholesterol crystals, there are several common substances and receptors sufficient to induce activation. It has been reported that ATP and various toxins including nigericin and maitotoxin could trigger the activation of the NLRP3 inflammasome via the purinergic 2X7 receptor (P2X7R), subsequently leading to ROS formation, potassium efflux, and mitochondrial DNA release [11–13]. However, ATP also exerts a negative regulatory effect in a rather indirect manner. When ATP binds to P2X7R, it also induces acetylcholine influx, restraining mitochondrial DNA release and NLRP3 activation via the *α*7 nicotinic acetylcholine receptor signaling pathway [13]. Interestingly, researchers also found that NLRP inflammasome formation occurs voluntarily when the cellular potassium level is below 90 mM and is suppressed at high potassium levels, indicating an indispensable role of K^+^ [14]. Under stimulation of oxLDL, CD36 helps to facilitate the intracellular conversion from soluble factors to crystals, thus causing lysosomal damage and rupture. Concomitantly, statistics also revealed that CD36-deficient macrophages generated much lesser IL-1*β* [15]. Other common crystals include silica and aluminum salts [16]. The overall scheme of NLRP3 inflammasome activation is presented in Figure 1.

## 3. Role of NLRP in Vascular Disease

### 3.1. Atherosclerosis

Characterized by endothelium dysfunction, foam cell formation, and lymphocyte infiltration, atherosclerosis is widely accepted to be a pathological process of inflammation [2]. However, the crucial role of the NLRP3 inflammasome in atherosclerosis was first proved by Duewell et al. [10]. They employed low-density lipoprotein receptor- (LDLR-) deficient mice with wild-type bone marrow or NLRP3^−/−^, ASC^−/−^, and IL-1*α*/*β*^−/−^ bone marrow. After 8 weeks of high fat diet, lower level of IL-18, an important biomarker of inflammasome, and attenuated atherosclerotic lesion were observed in the NLRP3^−/−^, ASC^−/−^, and IL-1*α*/*β*^−/−^ bone marrow group [10]. Consistently, caspase-1/11 depletion in bone marrow of the LDLR-deficient mice fed with high-fat diet also showed significant reduced atherosclerosis plaque [17].

In another study involving atherosclerosis-prone apolipoprotein E-null (Apoe^−/−^) mice, contradictory outcomes were observed. In 2001, researchers found that IL-18-binding protein, an IL-18 inhibitor, could not only attenuate the progress of plaque formation in the aorta but also decrease lymphocyte infiltration and lipid content in the lesion, thus exerting protective effects against atherosclerosis on Apoe^−/−^ mice [18]. Similarly, silencing IL-1*α*, IL-1*β*, and caspase-1 respectively in Apoe^−/−^ mice could attenuate atherosclerosis development [19–21]. However, Menu et al. reported contradictory results [22]. After analyzing NLRP3^−/−^, ASC^−/−^, or caspase-1^−/−^ on Apoe-null mice fed with a high-fat diet for 11 weeks, the team observed no significant change in cell infiltration, plaque stability, and atherosclerosis progression [22]. The mechanisms underlying such discrepancies remain undefined; however, Baldrighi et al. [1] suggested that this might be related to the duration of high-fat diet administration to the Apoe-null mice. Compared to mice with Apoe deficiency alone, Apoe^−/−^ mice fed with a high-fat diet and high-methionine diet (a hyperhomocysteinemia- (HHcy-) induced atherosclerosis model) showed an increased atherosclerotic plaque size. Silencing *NLRP3* in such models also reduced macrophage infiltration and HHcy-induced atherosclerosis lesions [23]. Noteworthily, in a transcriptomic analysis of human samples, we found that the mRNA level of NLRP3, ASC, caspase-1, IL-1*β*, and and IL-18 were all significantly increased in atherosclerotic plaque [24].

In addition to the fact that lacking components of NLRP inflammasomes or proinflammatory cytokines would probably exert protective effects against atherosclerosis, it is suggested that modulating correlative regulators could also exhibit similar effects. Increased P2X7 expression was observed in human atherosclerotic plaque and atherosclerosis-prone mouse models. Moreover, knocking down *P2X7* in Apoe^−/−^ mice delayed the progression of atherosclerosis [25]. Lectin-like oxLDL receptor-1 (LOX-1), a receptor for oxLDL, contributes to the lipid accumulation process of atherosclerosis. Several studies confirmed that in vivo deletion of *LOX-1* in LDLR^−/−^ mice fed with a high-fat diet for 18 weeks resulted in enhanced collagen deposition and attenuated atherosclerosis, while in vitro silencing of *LOX-1* in macrophages reduced mtDNA damage, ROS accumulation, and NLRP3 activation [26–28]. Given that mtDNA enrichment in the cytoplasm is detrimental, Tumurkhuu et al. [29] found a link between OGG1, an important DNA glycosylase that eliminates oxidized DNA, and atherosclerosis, possibly involving the NLRP3 inflammasome. Compared to LDLR^−/−^ mice fed with a western diet, OGG1^−/−^ LDLR^−/−^ mice displayed increased mtDNA accumulation, more severe inflammatory response, and larger atherosclerotic plaques. However, such phenomena could be reversed by silencing *NLRP3* [29], indicating that OGG1 is indeed a negative regulator of atherosclerosis. Furthermore, miR-9 has also been identified as a negative modulator that deactivates the NLRP3 inflammasome and reduces the atherosclerotic inflammatory response [30]. Although macrophages form a core component of atherosclerotic plaque and NLRP3 inflammasomes mostly reside in macrophages, recent evidence suggests that NLRP3 inflammasomes are also present in endothelial cells (ECs) [31]. EC dysfunction might be triggered by irregular blood flow or cytokines. Microparticles from macrophages induce the expression of adhesion molecules on ECs through the NLRP3 inflammasome, which later attract more inflammatory cells such as macrophages, thus forming an activation loop [31]. Sterol regulatory element-binding protein (SREBP) is a key regulator of cholesterol synthesis and an inducer of inflammation in ECs, which could provide both signals 1 and 2 for NLRP3 inflammasome formation [31]. When the activated form of SREBP was overexpressed in mice with Apoe deficiency, augmented atherosclerosis lesions were observed [31].

### 3.2. Others

Cholesterol crystals were found in both atherosclerosis plaques and the arterial wall of patients with abdominal aortic aneurysm (AAA). After conducting a genetic test of the NLRP3 inflammasome complex in more than 1000 AAA patients and control group, Roberts et al. reported a possible link between genetic variations of the NLRP3 inflammasome and the pathophysiology of AAA [32]. In a mouse model of coronary arteritis, Chen et al. found that inflammasome complex formation was hindered by silencing *NLRP3*, stabilizing the lysosome membrane or inhibiting cathepsin B, a critical factor released into the cytoplasm after lysosomal decomposition [33]. When mice were subjected to chronic exposure of aldosterone, vascular damage and elevated IL-1*β* level were observed. However, such effect could be abolished by silencing NLRP3, IL-1R, or caspase-1 [34]. Together, we can conclude that the NLRP3 inflammasome is critically involved in the pathological process of coronary arteritis as well as aldosterone-induced vascular damage [33, 34].

## 4. Role of NLRP in Ischemic Heart Disease

Considering that the heart tissue is susceptible to ischemia and has limited regenerative ability, reperfusion therapy has been employed in the treatment of ischemic heart disease. Reperfusion is typically accompanied by inflammation. On the one hand, inflammation is indispensable in wound healing and scar formation. On the other hand, excessive inflammatory response may result in adverse remodeling [35]. In 2011, Kawaguchi et al. examined cardiac tissues from patients who died of myocardial infarction (MI). Most of the infiltrated cells were found to be macrophages and neutrophils, showing a high expression of ASC [36]. Similarly, when wild-type (WT) mice and ASC^−/−^ mice were subjected to ischemia/reperfusion (I/R) injury, ASC^−/−^ mice showed a smaller infarcted area, decreased inflammatory cell infiltration, and improved cardiac remodeling. Given that inflammatory cell infiltration was not determined until 6 hours after I/R, an in vitro experiment revealed that the inflammatory response was activated in cardiac fibroblasts, but not cardiomyocytes, under the stimulation of LPS and that the underlying mechanism may involve cellular ROS generation and potassium efflux [36]. Moreover, expression of the NLRP3 inflammasome and its downstream inflammatory cytokines such as IL-1 and IL-18 was elevated in patients with coronary artery diseases or MI [37]. Consistently, Sandanger et al. [38] reported that the expression of the NLRP3 inflammasome and proinflammatory cytokines IL-1 and IL-18 was upregulated in mice that underwent MI surgery. Their data confirmed that inflammasome activation primarily existed in fibroblasts. When NLRP3^−/−^, ASC^−/−^, and WT hearts were exposed to ex vivo I/R injury, heart function was preserved and cell apoptosis was ameliorated in NLRP3^−/−^ hearts, but not in the ASC^−/−^ group [38]. However, contradictory results were presented later in 2016. Sandanger et al. observed an increased infarct size in NLRP^−/−^ mice 24 hours after I/R, while no significant difference was observed in lymphocyte infiltration [39]. When mice were pretreated with a cardioprotective compound before I/R, the due beneficial outcome was not observed in NLRP^−/−^ mice and ASC^−/−^ mice, suggesting a surprisingly protective role of the NLRP inflammasome in an I/R model [39]. The different timepoints chosen for assessment in these studies might account for this discrepancy, since the NLRP inflammasome was not abundantly activated in the early stage [40, 41]. Accordingly, when NLRP3 inhibitors were administered to the MI model, a decreased infarct size was seen at 24 hours after reperfusion, but not at 3 hours. Moreover, the beneficial effects were only observed when the NLRP3 inhibitor was injected immediately or 1 hour after reperfusion, and these effects were subverted when the NLRP3 inhibitor was injected 3 hours after reperfusion, indicating that pharmacological inhibition of NLRP3 has a limited therapeutic time window [41].

Apart from the well-known inflammatory damage induced by the NLRP3 inflammasome complex, caspase-1 has been reported to exert negative effects in an MI model by initiating pyroptosis, a type of programmed cell death in the form of cellular lysis. This damage was observed to be exacerbated in mice with a diabetic background [42]. Regarding the specific cell types in which inflammation occurs after I/R, Liu et al. recently identified cardiac microvascular endothelial cells to be involved, apart from fibroblasts [43]. NLRP3 inflammasome activation in endothelial cells is suggested to be mediated by thioredoxin-interacting/inhibiting protein (TXINP), indicated by the fact that employment of TXINP siRNA would disturb the formation of the NLRP3 inflammasome. Moreover, the NLRP3 inflammasome activation process is suggested to be ROS dependent [43].

## 5. Role of NLRP in Nonischemic Heart Disease

### 5.1. Diabetic Cardiomyopathy

DCM is a complication commonly observed at the terminal stage of diabetes; the inflammasome has been found to be pivotal in this process. Given that glucose is an essential inducer of the NLRP3 inflammasome, we speculate that the NLRP3 inflammasome may contribute to the pathological process of DCM [4]. In Luo et al.'s work [44], they employed a gene silencing approach to investigate the role of the NLRP3 inflammasome in type 2 diabetic rats fed with a high-fat diet. As expected, silencing *NLRP* attenuated cardiac inflammation, fibrosis, cell pyroptosis, and cardiac dysfunction in diabetic rats. Similar effects were noticed in H9C2 cells cultured in a high-glucose medium [44]. Moreover, ROS was found to be a critical mediator in inflammasome activation, as inhibiting ROS accumulation could abolish the activation of the NF-*κ*B and TXINP pathway and further diminish proinflammatory cytokine secretion [44].

### 5.2. Others

To further assess the role of the NLRP inflammasome in heart failure, Bracey et al. [45] constructed a mouse model in which the calcineurin transgene (CNTg) was heterozygously overexpressed specifically in the heart, mimicking chronic heart failure. Elevated *NLRP3* mRNA levels were observed along with cardiac hypertrophy, inflammation, and ventricular dilatation in CNTg mice. Concurrent with this finding, the team also discovered that genetic ablation of NLRP3 or administration of the IL-1 receptor antagonist could attenuate cardiac inflammation and rescue systolic dysfunction [45]. Moreover, a vital role of the NLRP3 inflammasome in a mouse model of hypertension has also been confirmed [46]. Li et al. performed transverse aortic constriction (TAC) in C57/BL6 mice to induce hypertension. Expression of the NLRP3 inflammasome and its downstream effectors was significantly increased in the TAC group, along with impaired cardiac function; triptolide could attenuate myocardial remodeling and improve cardiac function of the TAC mice by suppressing the NLRP3 inflammasome [46]. In addition, activation of the NLRP3 inflammasome and upregulation of IL-1 were observed both in cardiac fibroblasts pretreated with lipopolysaccharide (LPS) and in a sepsis mouse model [47]. Inhibition of the NLRP3 inflammasome by using glyburide could ameliorate myocardiac dysfunction induced by sepsis [47]. In the background of sepsis, Xie et al. found that *PKM2* knockout hindered the formation of the NLRP3 inflammasome and sepsis cell death [48]. In a coxsackievirus B3- (CVB3-) induced viral myocarditis model, the NLRP3 inflammasome, probably triggered by ROS and K^+^ efflux, was found to be critical in the pathogenesis process [49]. Surprisingly, in the aged heart, sustained increase in VEGF-A was found to exert negative effects on cardiac function. Such adverse impacts could be partially abolished by inactivating NLRP3, but not IL-1R or IL-18, indicating that NLRP3 may participate in regulating cardiac function independent of the inflammasome [50].

## 6. Possible Therapeutic Targets

Since it has been proven that the NLRP3 inflammasome is crucial in the pathological progression of various cardiac diseases, numerous experiments have been conducted to verify different approaches targeting either the NLRP3 inflammasome or its upstream regulators and downstream effectors. For example, in an Apoe^−/−^ mouse model fed with a high-fat diet, arglabin was demonstrated to effectively reduce the secretion of IL-1*β* and IL-18, convert proinflammatory M1 macrophage into anti-inflammatory M2 macrophage, induce autophagy, decrease the cholesterol level in plasma, and thus reduce the atherosclerosis size [51]. Moreover, in a randomized clinical trial, patients with coronary artery disease were administered atorvastatin or rosuvastatin for 8 months. Results showed that the expression level of the NLRP3 inflammasome was lower in the atorvastatin than in the rosuvastatin group. Such effects might be implicated in the slower progression of atherosclerosis [52]. Interestingly, rosuvastatin, on the other hand, was proven to be potent in the treatment of DCM by inhibiting the NLRP3 inflammasome and suppressing the MAPK pathway [53]. With regard to the beneficial effects of statin, Yu et al. proposed that statin might be utilized in the treatment of atherosclerosis and myocardial I/R injury, since it could regulate both the NLRP1 and NLRP3 inflammasomes [54, 55]. Furthermore, in vitro hypoxia-induced NLRP3 inflammasome activation in cardiomyocytes was abolished by the administration of pigment epithelium-derived factor (PEDF), a glycoprotein known to possess anti-inflammatory effects. PEDF is known to inhibit the NLRP3 inflammasome by eliminating mitochondrial damage and thus mtROS accumulation, the upstream regulator of inflammatory response [56].

In regard to the treatment of I/R injury, a novel small molecule named 16673-34-0 was identified as an effective pharmacological agent to reduce NLRP3 inflammasome activation in cardiomyocytes and decrease the infarct size in a mouse model of acute myocardial infarction (AMI) [57]. Similarly, colchicine was demonstrated to exert cardioprotective effects and improve the survival rate in an AMI mouse model, by inhibiting the mRNA expression level of NLRP3 inflammasome components [58]. Two clinical trials confirmed that colchicine is effective in reducing the infarct size in patients with ST-elevation MI and in decreasing cardiovascular events in patients with coronary disease [59, 60]. Apart from interfering with NLRP3 inflammasome assembly, blocking IL-1 function also serves as an alternative therapeutic approach [35].

The possible therapeutic targets are summarized in Table 1.

## 7. Conclusions

NLRPs are critical participants in the formation of inflammasomes and initiation of the immune response, which could be triggered by various DAMPs and PAMPs. While the indispensable role of inflammation was established in cardiovascular diseases such as atherosclerosis, aneurysm, cardiac ischemia/reperfusion injury, diabetic cardiomyopathy, chronic heart failure, and hypertension- or virus-induced cardiac dysfunction, the mechanism by which NLRP functions in these diseases has been widely investigated. Until date, we have deduced that the NLRP3 inflammasome contributes significantly to the pathological process of atherosclerosis, cardiac I/R injury, and other nonischemic cardiac diseases. In atherosclerosis and DCM models, silencing NLRP3 or other inflammasome components by using different approaches showed overall beneficial effects. However, outcomes from experiments manipulating the NLRP inflammasome in I/R models remain indefinite. In conclusion, targeting the NLRP inflammasome in cardiovascular disease treatment holds promise, and the optimization of therapeutic approaches requires further clarification regarding the precise role of NLRP in cardiovascular disease.

## Figures and Tables

**Table 1:** Possible therapeutic approaches targeting the NLRP3 inflammasome.

Experiment types	Model	Treatment	Effects	Ref.
Animal experiments or in vitro experiments	Apoe^−/−^ mouse model, high-fat diet	Arglabin	Reduced the secretion of IL-1*β* and IL-18, convert proinflammatory M1 macrophage into anti-inflammatory M2 macrophage, induce autophagy, decrease cholesterol level in plasma, reduce atherosclerosis size	[51]
	Type 2 diabetic rat model	Rosuvastatin	Inhibited the NLRP3 inflammasome and suppressed the MAPK pathway	[53]
	AMI mouse model	16673-34-0	Reduced the NLRP3 inflammasome activation in cardiomyocytes, decreased the infarct size	[57]
	AMI mouse model	Colchicine	Inhibited the mRNA expression level of NLRP3 inflammasome components, improved the survival rate	[58]
	In vitro hypoxia model	Pigment epithelium-derived factor (PEDF)	Inhibited the NLRP3 inflammasome by eliminating mitochondrial damage and thus mtROS accumulation	[56]

Clinical trials	Patients with coronary artery disease	Atorvastatin or rosuvastatin for 8 months	Reduced the expression level of the NLRP3 inflammasome and slowed the progression of atherosclerosis in the atorvastatin, but not the rosuvastatin, group	[52]
	Patients with ST-elevation MI	Colchicine	Reduced the infarct size	[59]
	Patients with coronary disease	Colchicine	Decreased the incidence of cardiovascular events	[60]
